# COVID-19 Mobile Positioning Data Contact Tracing and Patient Privacy Regulations: Exploratory Search of Global Response Strategies and the Use of Digital Tools in Nigeria

**DOI:** 10.2196/19139

**Published:** 2020-04-27

**Authors:** Iniobong Ekong, Emeka Chukwu, Martha Chukwu

**Affiliations:** 1 Department of Health Planning, Research and Statistics FCT Health and Human Services Secretariat Abuja Nigeria; 2 Department of Computer Information System Faculty of Information & Communication Technology University of Malta Msida Malta; 3 Ragnar Nurkse Department of Innovation and Governance School of Business and Governance Tallinn University of Technology Tallinn Estonia

**Keywords:** COVID-19, contact tracing, Nigeria’s National Data Protection Regulation, General Data Protection Regulation, GDPR, coronavirus, surveillance, mHealth, eHealth, digital health

## Abstract

**Background:**

The coronavirus disease (COVID-19) pandemic is the biggest global economic and health challenge of the century. Its effect and impact are still evolving, with deaths estimated to reach 40 million if unchecked. One effective and complementary strategy to slow the spread and reduce the impact is to trace the primary and secondary contacts of confirmed COVID-19 cases using contact tracing technology.

**Objective:**

The objective of this paper is to survey strategies for digital contact tracing for the COVID-19 pandemic and to present how using mobile positioning data conforms with Nigeria’s data privacy regulations.

**Methods:**

We conducted an exploratory review of current measures for COVID-19 contact tracing implemented around the world. We then analyzed how countries are using mobile positioning data technology to reduce the spread of COVID-19. We made recommendations on how Nigeria can adopt this approach while adhering to the guidelines provided by the National Data Protection Regulation (NDPR).

**Results:**

Despite the potential of digital contact tracing, it always conflicts with patient data privacy regulations. We found that Nigeria’s response complies with the NDPR, and that it is possible to leverage call detail records to complement current strategies within the NDPR.

**Conclusions:**

Our study shows that mobile position data contact tracing is important for epidemic control as long as it conforms to relevant data privacy regulations. Implementation guidelines will limit data misuse.

## Introduction

The coronavirus disease 2019 (COVID-19) is caused by severe acute respiratory syndrome coronavirus 2 (SARS-CoV-2) [1]. This infectious respiratory disease was first detected in Wuhan City, China, in December 2019. It was declared a global pandemic by the World Health Organization (WHO) on March 11, 2020, and has currently infected over two million people worldwide and has killed over 150,000 people. Globally, responses have been swift and in full influenza pandemic control mode [2]. Travel and movement restrictions to curtail spread both within and across cities are in force. Many cities around the world are in lockdown or lock-in mode. Some have issued dusk-to-dawn curfews. In other scenarios, large gatherings have either been banned or discouraged. Estimates suggest that this pandemic can claim the lives of as many as 40 million people globally [3]. The Spanish flu, which lasted between 1918 and 1920 in some places, has been estimated to have cost the lives of 21-50 million people globally [4]. Evidence suggests that influenzas can mainly be spread through large clusters [5]. The WHO global influenza preparedness plan presents guidelines for the management and control of influenza and other disease [6]. Nigeria, one of the countries that adopts WHO guidelines, has over 493 cases of COVID-19 as of April 17, 2020, with 17 mortalities. This is a substantial increase since the index case was reported on February 27, 2020. To better manage the spread, Nigeria’s federal government has declared a lockdown in key affected states (ie, Lagos, Ogun, and the Federal Capital Territory). The lockdown was in addition to several mitigating actions by state governments, ranging from a ban on social gatherings to dusk-to-dawn curfews. During the lockdown, schools, markets, churches, mosques, banks, offices, parks, motor parks, and airports remain closed, often for a 14-day period.

The Nigeria Centre for Disease Control (NCDC) reported that it is currently conducting contact tracing of over 9000 contacts of confirmed cases in an attempt to effectively contain the spread of the disease, in line with the recommended measures for pandemic response [7,8]. These measures include antiviral, vaccine, and nonpharmaceutical measures such as case isolation, household quarantine, school or workplace closure, and travel restrictions. Given the scale of the COVID-19 pandemic, nonpharmaceutical actions appear to be the only practical and logical option in the absence of any known antiviral drug or vaccine. Resources are stretched even in countries with advanced health care systems, as seen in Italy, the United Kingdom, China, and the United States [9,10].

Although the NCDC's approach has been commended for its compliance with WHO guidelines for large-scale containment and contact tracing, there remain options that may yet be explored [11]. Given the inadequacy of testing kits, it is believed that the number of confirmed cases may be far lower than the actual number of cases in Nigeria and most African countries. This is fueling speculations of a real, catastrophic-level pandemic if isolation, containment, quarantine, and contact-tracing mechanisms are not urgently implemented. In a country with an already weak health care system occasioned by poor health investment choices, managing such an outbreak will become impossible.

**Figure 1:**
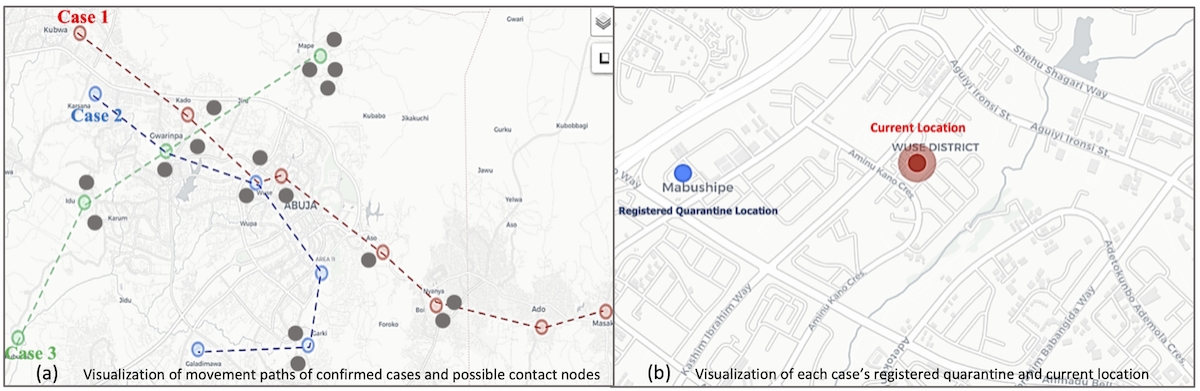
Visualization of Movement paths of cases and quarantine location.

There is, therefore, a need to develop and adopt new strategies, particularly digitally-enabled strategies, to facilitate a more extensive, accurate, seamless, and timely response in line with the high frequency of new infections among contacts of confirmed cases (ie, the secondary infection rate) [12]. The adoption of digital solutions in Nigeria has been focused on electronic forms for contact data collection and visualization for follow-up [13]. Digital technologies can do more than be a tool for field data collection or serve as an outbreak investigation platform. Data on households and general population movement patterns can be extracted through digital technologies [14]. Farrahi et al [15] showed that over a 9-month period, 72 participants made 10,992 phone calls and 9432 SMS records representing communication flow; additionally, these participants made 1,973,547 Bluetooth interactions representing physical proximity movements. When extrapolated for three cases in Abuja, the capital city of Nigeria, their movement can result in thousands of interactions. In the case of an infection, these three cases can initiate an exponential number of contacts through these interactions, as seen in Figure 1A. The registered quarantine address can be visualized on the map and movement of quarantine subjects can be monitored with notifications enabled (Figure 1B).

Our paper reviews global practices in the use of mobile positioning data to achieve a more targeted and efficient approach at contact tracing and disease surveillance, especially in the context of the COVID-19 pandemic. We discuss how this approach is possible within regulatory confines. We also recommend a novel strategy for coordinating agencies to leverage mobile positioning data, and how to ensure patient privacy is preserved.

## Methods

The COVID-19 pandemic is emerging and only three months old with little scholarly work to justify a systematic search, review, and analysis approach. We conducted an exploratory (nonsystematic) internet search for technology approaches and responses to COVID-19. Results from global and national agencies responsible for infection prevention and control were analyzed to ascertain how they currently use technology. We also reviewed how these use cases fit within the regulatory framework for contact tracing and isolation. A similar internet search methodology was adopted for Nigeria’s response and its use of digital tools for contact tracing.

## Results

Our search yielded results based on emerging trends and the use of digital technologies by countries around the world to respond to COVID-19. We first present global perspectives and response strategies on the use of mobile position data during previous and current pandemics. We then present Nigeria’s approach.

### Mobile Position Data: How It Works

The GSM Association puts the total number of mobile subscribers at 5 billion unique subscribers and 7 billion connected devices [16]. Nigeria has 184 million active mobile subscriber lines [17]. Mobile telecommunications subscriber communication and movement data was used for contact tracing during the Ebola outbreak [14]. Many countries are currently using mobile data for a more rapid response to the COVID-19 pandemic [18,19]. There has been a 90% increase in the number of countries implementing digital tracking measures and a 100% increase in reports of censorship [20]. These approaches range from the use of anonymized aggregate data to monitor the general mobility of people and track the mobile phones of confirmed cases to tracking suspected patients and their contacts. In some cases, these approaches were individualized and mandatory while, in others, they were aggregated and anonymized. In all cases, there were collaborations between the government, mobile network operators (MNOs), and other data controllers such as technology companies and financial services providers.

**Figure 2:**
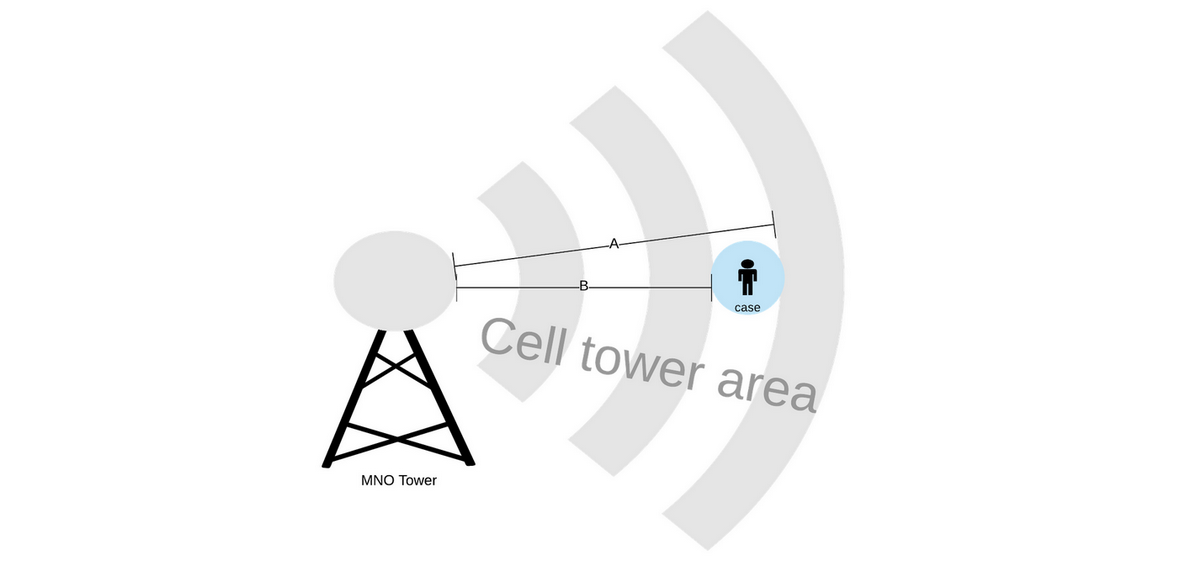
Location of a subject with respect to a mobile network operator (MNO) cell tower.

At any time, each mobile subscriber is connected to a segment of the MNO base station tower. For simplicity, we have presented a cell tower and a subscriber in Figure 2. We used letters A and B to illustrate the farthest and shortest distance of the subscriber from the base station tower based on power throughput and internal cell tower position triangulation. The difference between A and B, representing the diameter of a user’s device, which is a proxy for the user’s location, often ranges between 50 and 300 meters, but depends other factors [14].

### Global Strategies

Table 1 details some of the strategies governments around the world are adopting to track and isolate COVID-19 patients and their contacts or for lockdown/lock-in enforcement. In the United States, US $500 million of the US $2 trillion economic stimulus bill recently signed into law has been allocated to the US Centers for Disease Control and Prevention to launch a new surveillance and data collection system to monitor the spread of COVID-19 [21]. This move is a first for the United States since stringent patient data privacy and security regulations have hampered the adoption of contact tracing as a countermeasure for epidemic control in the past [22]. Similarly, the state of Massachusetts has announced the launch of what it calls the “first contact tracing” call center with 1000 virtual assistants to call and trace contacts of COVID-19-positive persons [23].

The European Union’s General Data Protection Regulation (GDPR) is being tested on a large scale. Within the regulation, a patient can decide not to disclose who they have been in contact with or legally resist being traced [24]. Evidence has emerged that Germany, Austria, and Italy are using aggregated call detail records (CDRs) to enforce lockdown and stay-at-home policies [25]. As this is an evolving challenge and European countries such as Italy and France are amongst the worst affected, changes to the GDPR regulations are expected and anticipated.

In China, the government worked with telecommunications companies to track and contact people who had traveled through Hubei province during the early days of the disease outbreak. Location data was shared with China's National Health Commission and other agencies, enabling them to retrospectively simulate the location of confirmed cases and their contacts, who were then issued warnings via social media [26]. Information has also emerged that the Chinese government may have leveraged its large network of sensors and surveillance cameras supported by an artificial intelligence–powered facial recognition and recommender system in its response to the COVID-19 outbreak [27]. This success may not be unconnected with the often criticized and loose patient data privacy and security regulation in China.

It was, however, observed that the extent of compliance with international and country-level regulations regarding data privacy considerations in deploying this digital technology varied from country to country.

**Table 1:** Strategies planned or adopted by countries for the use of mobile positioning data in response to the COVID-19 pandemic.

Country	Strategy planned or adopted
United States [21]	The state of Massachusetts announced the launch of its first contact tracing call center to be manned by 1000 virtual assistants [23]. The US federal government announced a US $500 million package for COVID-19 surveillance for the CDC [21].
China [22,28]	A mandatory smartphone app “Health code” that leverages a mesh network for infected persons contact tracing and notification.
Italy, Germany, and Austria [25]	Telecommunications providers allow for the sharing of location data with health authorities to check whether people are remaining at home. The data is aggregated and anonymous, mapping concentrations rather than individuals to respect Europe’s privacy laws.
South Korea [29]	The government created a map of cell phone data provided by telecommunications and credit card companies. The map was made public, so everyone could track their level of exposure.
Israel [19]	The government is using GMS call detail records in addition to patient mobile phone position data to locate contacts and trace their movement patterns.
Iran [30]	Iranian authorities developed a mobile app with government endorsement for COVID-19 self-diagnosis checks. It, however, also discretely collects user’s location data.
Singapore [18,21]	Singapore is using a mobile app that uses a Bluetooth-based mesh network to detect people's proximity to those who have been exposed to COVID-19 and warns them to get tested if they come in close contact.

### The Nigerian Strategy

Human travel patterns and mobility can be assessed using available mobile phone data, and its application can be useful in disease epidemiology [31]. Panigutti et al [31] also revealed the adequacy of mobile phone data for tracking infectious disease spread, particularly in heavily populated and highly interconnected communities.

Border restrictions, internal travel restrictions, and school closures or total lockdown are reasonable but have minimal impact compared to effective case isolation or quarantine, which have been shown to have a significant impact if properly conducted [2]. This is particularly important in Nigeria’s case, where total compliance to these strategies cannot be guaranteed. Therefore, data on case isolation and quarantine should be a significant priority in Nigeria. Moreover, data is useful in modeling disease transmission. Specifically, collecting and analyzing data on transmission in different social contexts is highly effective in mapping intervention strategies since the impact of case isolation and quarantine depends on reducing contact between unaffected individuals and the index and other cases while they are ill [2].

In order for the NCDC to effectively conduct the current large-scale contact tracing of over 9000 contacts of confirmed cases, use of digital technology is inevitable. The number of contacts may even be more than this number considering the frequency of new infections. Currently, there are several digital contact data capture solutions, including the *Surveillance, Outbreak Response Management and Analysis System* (SORMAS). These solutions require a field epidemiologist or their representative to visit every contact.

## Discussion

### Principal Findings

Evidence suggests that contact tracing and data protection can go together [32]. Significant progress is being made with current strategies. As promising as they may seem, data privacy concerns remain a major impediment; it is necessary to find a balance between deploying the technology and maintaining data safety and patient privacy. Existing patient privacy regulations are currently being tested. Some countries have attempted to relax existing stringent regulations that protect patient privacy to allow for greater access; others have worked around them. According to Woods [21], many of the new digital technology approaches appear inevitable and legitimate, given the unprecedented high frequency of the COVID-19 infection spread. Many countries have now also invoked speedy legislative processes to give legitimacy to their workarounds and deployments.

In Israel, for instance, the cabinet has passed an emergency law to use mobile data for tracking people infected with COVID-19, trace their contacts, and identify individuals for quarantine [19]. This law was passed overnight, bypassing parliamentary approval. In the United States, privacy advocates are proposing stringent procedures to keep personal information safe, including deletion, once the data are no longer in use to prevent abuse by law enforcement agents [21].

The National Data Protection Regulation (NDPR) of Nigeria was promulgated in 2019 [33]. Amongst other stipulations, the regulation outlines the guiding principles for data processing in Section 5. These principles consider data processing unlawful if there is no consent by the individual data subjects (in this case, the confirmed persons), if it is inaccurate with prejudice to human dignity and not protected against cybercrime, and if it is stored beyond a reasonable period of time. However, despite these guiding principles, Section 6, part 2.0, subsection 2.2 (e) of the document lists the conditions for lawful data processing and states that:

Processing is necessary for the performance of a task carried out in the public interest or the exercise of official public mandate vested in the controller.

The data controller in the case of mobile positioning data is the MNO, the entity that determines the purposes for and the manner in which network subscriber phone data is processed or is to be processed. Section 11 of the regulation states that data processing by a third party (eg, a public authority such as the Federal Ministry of Health, the NCDC, or anybody engaged in processing the location data such as a technology company) shall be governed by a written contract with the data controller. Interestingly, though the NDPR protects the privacy of personal mobile location data, it also provides exceptions for the use of such data that override public interest, such as the current COVID-19 outbreak.

### Recommendations

Mobile phone location data can be effectively utilized in Nigeria for COVID-19 response. The government can leverage existing mobile technology resources and infrastructure available in-country by working with MNOs and technology firms to optimize the ongoing contact tracing and surveillance of over 9000 known contacts of confirmed cases. This collaboration should be guided by the NDPR in order to protect and safeguard individuals' data, prevent a breach of data privacy rights as well as inappropriate use and abuse by law enforcement agencies beyond the period of contact tracing and surveillance.

In practice, however, the first step should involve anonymized mobile subscriber data in line with good data governance policy. In the spirit of goodwill, informed consent of confirmed cases should be appropriately obtained once they are diagnosed, whenever possible. The use of the public interest exception should be a last resort. A simplified guideline for these processes for adhering to the NDPR should be written and made transparently available for data custodians, requesting bodies, data handlers, and the patient or contact.

A third-party agreement should also be formally signed between parties interfacing with patient data in any way. A typical use case sensitive to data privacy concerns is the use of information only regarding visits to public facilities, including public transportation systems, parks, churches, mosques, or malls, by COVID-19-positive individuals, as described by Ohmukai et al [34]. The use of CDRs has been proven to be effective in detecting outbreak clusters, followed by the use of other frontline data collection tools for mitigating impact and containment [14]. A key limitation of using CDRs from MNOs is that for basic 2G (second generation) phone users, the location will rely on mobile network phone mast location triangulation only. This approach alone has a proximity accuracy of 50-300 meters. This accuracy level is not sufficient to identify persons who have been in contact with a COVID-19 patient since the WHO contact definition prescribes two meters [7]. The use of telecommunication CDRs should complement other strategies for effective results.

The immediate action after a successful contact trace is communicating the expected course of action to citizens of an infected community cluster. A simple, user-friendly interface using Unstructured Supplementary Service Data will help improve information requests and management for low-income but literate users. Interactive voice response technology will be suitable and appropriate for awareness response for low-literate users in their local language.

### Conclusions

Mobile positioning data can significantly improve the capacity and scope of timely outbreak response and will help governments as well as other responders in Nigeria. When implemented early [15], there are opportunities to leverage positioning data to break the chains of disease transmission in community clusters. It can improve the efficiency of currently used field data collection and outbreak investigation platforms when used in synergy.

While mobile positioning data can be used within the current regulation, guidelines for data handlers must include measures to curtail misuse and unauthorized access. Future research should design and implement models for mobile position contact tracing.

## Abbreviations

2G: second generation
CDC: US Centers for Disease Control and Prevention
CDR: call detail record
COVID-19: coronavirus disease
GDPR: General Data Protection Regulation
MNO: mobile network operator
NCDC: Nigeria Centre for Disease Control
NDPR: National Data Protection Regulation
SARS-CoV-2: severe acute respiratory syndrome coronavirus 2
SORMAS: Surveillance, Outbreak Response Management and Analysis System
WHO: World Health Organization
